# Management of Neuromuscular Blocking Agents in Critically Ill Patients with Lung Diseases

**DOI:** 10.3390/jcm13041182

**Published:** 2024-02-19

**Authors:** Ida Giorgia Iavarone, Lou’i Al-Husinat, Jorge Luis Vélez-Páez, Chiara Robba, Pedro Leme Silva, Patricia R. M. Rocco, Denise Battaglini

**Affiliations:** 1 Anesthesia and Intensive Care, IRCCS Ospedale Policlinico San Martino, 16132 Genova, Italy; idagiorgia.iavarone@gmail.com (I.G.I.); kiarobba@gmail.com (C.R.); 2 Department of Surgical Sciences and Integrated Diagnostics, University of Genova, 16132 Genova, Italy; 3 Department of Clinical Sciences, Faculty of Medicine, Yarmouk University, Irbid 21163, Jordan; loui.husinat@yu.edu.jo; 4 Facultad de Ciencias Médicas, Universidad Central de Ecuador, Quito 170129, Ecuador; jorgeluisvelez13@hotmail.com; 5 Unidad de Terapia Intensiva, Hospital Pablo Arturo Suárez, Centro de Investigación Clínica, Quito 170129, Ecuador; 6 Laboratory of Pulmonary Investigation, Carlos Chagas Filho Institute of Biophysics, Federal University of Rio de Janeiro, Rio de Janeiro 21941, Brazil; pedroleme@biof.ufrj.br (P.L.S.); prmrocco@gmail.com (P.R.M.R.)

**Keywords:** neuromuscular blocking agents, intensive care unit, acute respiratory distress syndrome, status asthmaticus, chronic obstructive pulmonary disease

## Abstract

The use of neuromuscular blocking agents (NMBAs) is common in the intensive care unit (ICU). NMBAs have been used in critically ill patients with lung diseases to optimize mechanical ventilation, prevent spontaneous respiratory efforts, reduce the work of breathing and oxygen consumption, and avoid patient–ventilator asynchrony. In patients with acute respiratory distress syndrome (ARDS), NMBAs reduce the risk of barotrauma and improve oxygenation. Nevertheless, current guidelines and evidence are contrasting regarding the routine use of NMBAs. In status asthmaticus and acute exacerbation of chronic obstructive pulmonary disease, NMBAs are used in specific conditions to ameliorate patient–ventilator synchronism and oxygenation, although their routine use is controversial. Indeed, the use of NMBAs has decreased over the last decade due to potential adverse effects, such as immobilization, venous thrombosis, patient awareness during paralysis, development of critical illness myopathy, autonomic interactions, ICU-acquired weakness, and residual paralysis after cessation of NMBAs use. The aim of this review is to highlight current knowledge and synthesize the evidence for the effects of NMBAs for critically ill patients with lung diseases, focusing on patient–ventilator asynchrony, ARDS, status asthmaticus, and chronic obstructive pulmonary disease.

## 1. Introduction

Neuromuscular blocking agents (NMBAs) represent a landmark in modern anesthesia, acting on the neuromuscular junction by blocking the transmission of nervous impulses in the motor endplate of striated muscles, resulting in skeletal muscle paralysis [1].

The use of NMBAs is common in the intensive care unit (ICU), especially in cases of acute distress respiratory syndrome (ARDS). It is used in 25–45% of cases, with different practices associated with geographic differences [2]. NMBAs are used in pulmonary critical care patients, such as those with ARDS, to optimize mechanical ventilation (MV), prevent spontaneous respiratory efforts, reduce the work of breathing and oxygen consumption, reduce the risk of barotrauma, and avoid patient–ventilator asynchrony [3,4]. NMBAs have many other beneficial effects on lung function, improving alveolar recruitment, and they can reduce the concentration of interleukins and tumor necrosis factor-alpha, leading to anti-inflammatory effects [5].

Patients with severe ARDS, status asthmaticus, and chronic obstructive pulmonary disease (COPD) often need MV support, which is frequently insufficiently controlled with sedative and analgesic drugs [2,6,7]. NMBAs seem to have beneficial effects on airway pressures. In a small trial conducted on mechanically ventilated children with severe acute hypoxemic respiratory failure, NMBAs decreased the mean airway pressure (*p* = 0.039) and the oxygenation index (OI) (*p* = 0.039) in all patients [8]. In a recent trial conducted on 30 patients with moderate-to-severe ARDS, neuromuscular blockade treatment did not affect the transpulmonary driving pressure (expressed as inspiratory lung pressure minus expiratory lung pressure and defined as a surrogate of the stress applied to the lungs) at 48 h [9]. NMBAs also seem to play a role in gas exchange. In their study, Gainnier et al. [10] reported a higher PaO_2_/FiO_2_ ratio at 48, 96, and 120 h in patients randomized to the NMBA group (*p* = 0.021).

Thus, when deep sedation fails or is not tolerated, NMBAs could be administered to harmonize the respiratory function [4].

Although these beneficial effects, especially in patients with ARDS, the impact of NMBAs on mortality remains controversial [11]. The routine use of NMBAs in ICUs has decreased in the last decade due to potential harmful effects resulting from immobilization such as venous thrombosis, development of critical illness myopathy, ICU-acquired weakness (ICUAW), autonomic interactions, awareness during paralysis, and residual paralysis after cessation of NMBAs [4,12]. However, the real benefits and complications of NMBAs in critically ill patients with lung diseases have not been completely elucidated.

The aim of this review is to highlight current knowledge and synthesize the evidence concerning the effects of NMBAs in critically ill patients with lung diseases, particularly in cases of patient–ventilator asynchrony, ARDS, status asthmaticus, and COPD.

## 2. Methods

We searched PubMed, MEDLINE, Embase, and Scopus for observational studies, randomized controlled trials, meta-analyses, and current guidelines evaluating the administration of NMBAs in critically ill patients with lung diseases (ARDS or status asthmaticus or COPD).

## 3. Classification of NMBAs, Pharmacokinetics and Pharmacodynamics

Neuromuscular blockade acts at the neuromuscular junction. When an electric impulse is released in the motor neuron, acetylcholine (ACh) is accumulated in vesicles of the presynaptic membrane acting on the nicotinic receptors on the postsynaptic membrane and causing muscle contraction [13]. Pharmacokinetics and pharmacodynamics of the most commonly used NMBAs are reported in Table 1.

Besides the neuromuscular blockading action, NMBAs have an anti-inflammatory effect [14]. Particularly in patients with ARDS, NMBAs decreased the pro-inflammatory response [15], as well as the levels of biomarkers associated with epithelial and endothelial lung injury [16].

Recently, a new series of neuromuscular complexes called the chlorofumarates (gantacurium, CW002, and CW011) are being developed with a promising pharmacodynamic profile; however, availability for clinical use remains undefined. Other studies are required to establish the role of these drugs in clinical practice [17].

**Table 1:** Pharmacokinetics and pharmacodynamics of the most commonly used NMBAs.

Agent	Duration	ED95 (mg/kg)	Onset Time (min)	Duration (min)	Dosing	Metabolism
Depolarizing *						
Succinylcholine	Ultra-short	0.3	1–1.5	5–10	1 mg/kg bolus NA	Plasma cholinesterase
Non-Depolarizing **						
Aminosteroids						
Rocuronium	Intermediate-duration agent	0.3	1.5–3	20–35	0.6–1.2 mg/kg bolus 8–12 mcg/kg/min infusion	Hepatic, no active metabolites
Vecuronium	Intermediate-duration agent	0.05	3–4	20–45	0.08–0.1 mg/kg bolus 0.8–1.7 mcg/kg/min infusion	Hepatic, bile, urinary metabolites
Pancuronium	Long-duration agent	0.07	2–4	60–100	0.05–1 mg/kg bolus 0.8–1.7 mcg/kg/min infusion	Renal elimination
Benzylisoquinolines						
Cisatracurium	Intermediate-duration agent	0.05	5–7	30–60	0.1–0.2 mg/kg bolus 1–3 mcg/kg/min	Hoffmann reaction, renal elimination
Atracurium	Intermediate-duration agent	0.2–0.25	3–4	20–35	0.4–0.5 mg/kg bolus 5–10 mcg/kg/min	Hoffmann reaction, plasmatic esterase
Mivacurium	Short-duration agent	0.08	3–4	15–20	0.15–0.25 mg/kg bolus 9–10 mcg/kg/min infusion	Plasmatic esterase
Doxacurium	Long-duration agent	0.025	5–10	40–120	0.03–0.06 mg/kg NA	Renal elimination
Chlorofumarate diesters						
Gantacurium	Ultra-short duration agent	0.19	1.7	6–8	0.2–0.5 mg/kg NA	Addition of cysteine and ester hydrolysis

* Depolarizing NMBA causes depolarization of the postsynaptic membrane, resulting in resistance to the activity of acetylcholine [18]. ** Non-depolarizing NMBAs compete with acetylcholine for the binding site on the alpha subunit of the nicotinic receptors, preventing its action and establishing a neuromuscular blockade [19]. NA, not available.

## 4. General Advantages and Disadvantages of Using NMBAs in Critically Ill Patients with Lung Diseases

NMBAs can ameliorate the management of ventilation [20], limiting decruitment, inspiratory effort, and expiratory alveolar collapse [9]. Some studies demonstrated improved oxygenation using NMBAs, possibly related to the effects on reducing the work of breathing [12,21]. In a randomized controlled trial on patients with ARDS receiving conventional therapy plus placebo or NMBAs, treatment with cysatracurium exerted anti-inflammatory effects by reducing the concentration of interleukins and tumor necrosis factor-alpha in serum and bronchoalveolar lavage [5].

Intra-abdominal hypertension (IAH), defined as an intra-abdominal pressure (IAP) above 12 mmHg, is one of the possible conditions in which the use of NMBAs can improve lung function. It is estimated that around 20% of patients present with IAH on admission to the ICU and almost 50% will develop IAH within the first week in the ICU [21,22]. IAH often progresses with an upper shift of the diaphragm and decreased lung volume and chest wall compliance, resulting in increased airway pressures [23] and decreased oxygenation [24]. Although abdominal contractions can falsely increase IAP values, to date, no recommendation on increasing sedation or using NMBAs to accurately measure IAP has been defined [24]. A recent guideline for the management of IAH and abdominal compartment syndrome in critically ill patients highlighted the possibility of considering the use of NMBAs for persistent IAH [25].

When paralyzing the patient, it is always important to consider the possibility of the development of complications associated with the administration of NMBAs, such as corneal abrasions [4] and venous thrombosis [26], and complications associated with prolonged immobilization such as ICUAW and myopathy. The relationship between ICUAW and NMBAs is controversial [4]. Although a recent meta-analysis did not show an association between NMBAs and neuromuscular dysfunction acquired in critical illness (odds ratio (OR), 1.21; 95% confidence interval (CI), 0.67–2.19), merged data from all the included studies suggested a modest association (OR, 1.25; 95% CI, 1.06–1.48; I = 16%) between NMBA use and ICUAW [27]. Many other studies have confirmed the association [28,29] or the potential risk [30] of the development of ICUAW with the use of NMBAs, but with a weak study design and high risk of bias because of the multi-factorial causes of ICUAW and heterogeneous outcomes [31]. In this uncertainty, the association between the use of NMBAs and critical weakness does not seem to be reasonable. Thus, recent SCCM guidelines did not relate the use of NMBAs with the risk of ICUAW, rather associating it with prolonged immobility and muscle disuse [32]. In addition, NMBAs impaired airway protective reflexes [33] and increased the risk of upper airway obstruction and pneumonia. Moreover, these patients needed deep sedation due to prolonged treatment with NMBAs [34].

Critically ill patients often have multi-organ-system disorders and receive treatments for longer periods; thus, the elimination of NMBAs and metabolites can be delayed, resulting in greater accumulation [4,35] and adverse events, difficulty in weaning from the ventilator [36], and the risk of venous thrombosis [26].

## 5. Patient–Ventilator Asynchrony

Patient–ventilator asynchrony is frequently observed during MV and is associated with worse outcomes and higher mortality [37].

Ventilatory under-assistance or over-assistance translates to different types of asynchronies [38]. Under-assistance could lead to an increased load on respiratory muscles, air hunger, and lung injury caused by excessive tidal volumes (V_T_). Over-assistance could yield decreased inspiratory drive, which may result in reverse triggering, thus worsening lung injury. In addition, asynchronies may increase intrathoracic pressure, thus modifying cardiac output and hemodynamic status [39].

Yoshida et al. [40] demonstrated that an increase in distending pressure, caused by spontaneous effort in mechanically ventilated patients, could worsen a pre-existing lung injury through a pendelluft effect from non-dependent lung areas toward dependent areas because the diaphragm contraction is poorly transmitted across the pleural surface in an injured lung. Therefore, management of patient–ventilator asynchrony with neuromuscular blockade may be considered to minimize the lung and diaphragm injury associated with spontaneous breathing [41], especially in patients with ARDS [42].

The use of NMBAs in the critical care setting is frequently guided by personal experience and local practice, more than validated guidelines and recommendations [32]. NMBAs minimize the risk of ventilator-induced lung injury (VILI). However, the use of NMBAs requires adequate sedation to prevent VILI and may lead to extended time on MV, longer ICU stays, and increased risk of ventilator-associated pneumonia (VAP) [43,44]. NMBAs should be administrated with adequate sedation. Nevertheless, the sedation level is a factor that could affect the incidence of asynchrony. Observational studies showed an association between deep sedation and a higher incidence of patient–ventilator asynchronies [37,45].

A multi-center study showed a lower incidence of asynchronies with lighter sedation with dexmedetomidine compared with deeper sedation with propofol [46]. So, increasing sedation does not always represent an effective strategy to reduce asynchrony. When asynchrony is related to double triggering, deeper sedation associated with neuromuscular blockade could be taken into consideration. In contrast, in the case of reverse triggering, muscle effort could result in inflation so that a reduced sedation and NMBA strategy could be considered [47].

## 6. Acute Respiratory Distress Syndrome

To date, pharmacologic therapies have shown no beneficial effects in patients with ARDS, but supportive treatments such as MV can improve ARDS outcomes [48]. NMBAs have been largely used in patients with ARDS over the years [49], given that they can minimize VILI in the presence of increased respiratory drive or patient ventilatory asynchrony [50]. Lighter sedation and an early active breathing strategy are increasingly used for patients with ARDS to reduce muscle wasting [3,51,52,53]. Therefore, the use of NMBAs in this population is controversial.

Many trials focusing on the use of NMBAs have been conducted on patients with ARDS (Table 2), but no consensus has been reached, and specific recommendations are currently being formulated.

Controversial results were shown concerning the mortality rate in two larger studies: the ACURASYS and ROSE trials [52,54].

The ACURASYS trial reported a reduction in mortality in patients with moderate to severe ARDS; in contrast, the ROSE trial did not find significant changes in mortality. The differences between these two trials may be attributed to certain factors. (1) Differences in the definition of ARDS: even though, in both studies, patients presented PaO2/FiO2 < 150 mmHg, in the baseline of the ROSE trial, positive end-expiratory pressure was higher (≥8 cm H2O) [52]. (2) The enrollment of patients was later in the ACURASYS trial (16 h) compared with the ROSE trial [54] (8 h), resulting in different study populations and potential bias. (3) Pharmacologic treatments differed between the studies. (4) In the ROSE trial [52], a lighter sedation strategy was used in the control group, whereas in the ACURASYS trial [54], deep sedation was used in both the treatment and placebo groups. (5) Although both studies used protective lung ventilation strategies, in the ROSE trial, a lower FiO2 was applied, but PEEP was higher and tidal volume was lower in both study arms [52].

Some meta-analyses showed improvement in oxygenation and reduction in barotrauma risk in patients with ARDS treated with NMBAs [55,56,57,58]. These controversial results were also confirmed in a recent analysis of the administration of NMBAs in cases of ARDS [59]. In contrast, another recent meta-analysis of five trials endorsed by the European Society of Intensive Care Medicine (ESICM) found no significant effect on outcomes and 28-day mortality in patients with ARDS treated with NMBAs compared with patients with ARDS who were not treated [14,52]. These controversial results may be associated with high data heterogeneity. In addition, Plens et al., in a recent study, demonstrate that NMBA infusion during ARDS could reduce expiratory muscles activity and increase end expiratory lung volume leading to a benefit in MV [60].

During the COVID-19 pandemic, NMBAs were frequently administered in patients with ARDS to reduce spontaneous efforts and thus transpulmonary pressures [53]. To date, no randomized controlled trials using NMBAs in patients with COVID-19 ARDS have been published [53,54]. A recent study observed a reduction in the duration of MV and mortality in patients with COVID-19 ARDS treated with NMBAs [61]. However, in a study conducted on 1953 patients with COVID-19 and moderate/severe ARDS, early and short courses of NMBAs did not reduce 90-day mortality and ventilator-free days [62]. The 2017 ESICM clinical practice guideline did not investigate NMBAs in the treatment of ARDS because of resource constraints [63]. More recent guidelines concluded that there is no evidence to support the routine use of NMBAs in cases of ARDS [32]. The ESICM guidelines on ARDS, published in 2023 [53], recommend against the routine use of continuous infusions of NMBAs to reduce mortality in patients with moderate/severe ARDS with a strong recommendation and a moderate level of evidence. Furthermore, because of the lack of evidence, the routine use of continuous infusions of NMBAs in patients with ARDS due to COVID-19 was not recommended [53]. In contrast, an update of the American Thoracic Society guidelines suggests neuromuscular blockade in patients with early (≤48 h from MV therapy) severe ARDS (PaO2/FiO2 ≤ 100) [64]. In short, clinical evidence suggests that NMBAs might be considered in selected cases with early and severe ARDS with deep sedation, invasive MV, and the need for prone positioning within 48 h [32,56]. The use of NMBAs must be individualized, and further studies are required [4]. Two new trials investigating the use of cisatracurium in cases of moderate/severe ARDS are ongoing: (1) a comparison between bolus and continuous infusion (NCT05153525); and (2) early NMBAs versus sedation alone (NCT04922814). Another trial, which titrated NMBAs in spontaneous breathing patients with severe ARDS (partial neuromuscular blockade in acute respiratory distress syndrome (PNEUMA)) supported with venovenous extracorporeal membrane oxygenation recently finished, but no results have been published.

## 7. Status Asthmaticus

Status asthmaticus is a severe, persistent asthma attack that does not respond to usual treatments; it is characterized by hypoxemia, hypercapnia, and secondary respiratory failure [65]. A retrospective review reported that 61.2% of patients hospitalized for status asthmaticus required intubation and MV [7]. In the case of deterioration of respiratory conditions, despite initial pharmacologic treatment, intubation and MV are required. In addition, when patient–ventilator asynchronies, hypoxemia, or dynamic hyperinflation occur, even with deep sedation, the risk of generating auto-PEEP or barotrauma is high, thus requiring NMBAs [66], which then improve oxygenation and hemodynamics.

In an analysis of 30 years of ICU admissions for status asthmaticus, the use of NMBAs in mechanically ventilated patients with status asthmaticus has increased in the last 10 years [7], but this therapy remains controversial.

In a retrospective large study, Adnet et al. [28] analyzed the morbidity of intubated asthmatic patients receiving long-term (>12 h) NMBAs and found that VAP, post-intubation myopathy, and duration of ICU stay were higher in the group of patients treated with NMBAs.

Peters et al. [7] reported similar findings and an equivalent overall rate of myopathy incidence in patients with status asthmaticus receiving NMBAs. In contrast, Kesler et al. [29] demonstrated that the risk of myopathy in status asthmaticus was not associated with the duration of NMBAs because patients who underwent a short period of neuromuscular blockade also developed weakness. Replacing NMBAs with a continuous deep sedation strategy did not seem to modify the incidence of muscle weakness in patients with status asthmaticus. A recent paper from Qiao et al. [67] evaluated the risk of rhabdomyolysis, a rare but potentially fatal complication, in patients with status asthmaticus treated with high doses of steroids or theophylline combined with NMBAs, thus enhancing the debate on the use of NMBAs in status asthmaticus.

Current knowledge and the 2016 guideline for sustained neuromuscular blockade in critically ill patients suggest against the routine administration of NMBAs to mechanically ventilated patients with status asthmaticus [32].

## 8. Chronic Obstructive Pulmonary Disease

COPD is a heterogeneous lung condition characterized by chronic respiratory symptoms (dyspnea, cough, expectoration, and/or exacerbations) due to abnormalities of the airways (bronchitis, bronchiolitis) and/or alveoli (emphysema) that cause persistent, often progressive, airflow obstruction [68]. COPD is characterized by expiratory flow limitation, resulting in air trapping and dynamic hyperinflation, leading to auto-PEEP, increased intrathoracic pressure, and breathing efforts, as well as the risk of barotrauma. Acute respiratory failure due to an exacerbation of COPD has been associated with severe respiratory acidosis, increased levels of dyspnea, muscle fatigue, compromised neurologic status, and hemodynamic instability [6], which may require MV, either invasive or non-invasive. Sedation and occasionally paralysis with NMBAs may be needed to decrease patient–ventilator asynchrony [68,69].

In the ICU, half of patients with COPD are considered difficult to wean from MV [70]. As already described for status asthmaticus [28,29,65,71], weaning failure has been attributed to muscle weakness caused by a combination of NMBAs and corticosteroids [72]. In addition, the continuous administration of NMBAs and high doses of sedatives contribute to muscle atrophy [73]; thus, it is recommended that they are used for as short a time as possible [72]. The occurrence of respiratory muscle dysfunction caused by NMBAs may further worsen the respiratory pump performance in patients with COPD [72].

## 9. Monitoring of Neuromuscular Blockade and Adequacy of Sedation

Neuromuscular monitoring is indispensable for optimal management of NMBAs [35]. A peripheral nerve stimulator was introduced in the 1950s and is useful for monitoring neuromuscular blockade. In 1970, Ali et al. [74] reported train-of-four (TOF) testing to measure the degree of neuromuscular blockade through the use of a peripheral nerve stimulator. The goal of TOF monitoring is to ensure that the minimum amount of NMBA is administered to adequately paralyze the patient. TOF stimulation releases four electrical pulses to a peripheral nerve. The pattern involves stimulating the ulnar nerve with a TOF supramaximal twitch stimuli with a frequency of 2 Hz, i.e., four stimuli each separated by 0.5 s. The TOF is then repeated every 10 s (train frequency of 0.1 Hz). As well as enabling the observer to compare T1 (first twitch of the TOF) to T0 (control), it also enables comparison of T4 (fourth twitch of the TOF) to T1. This is known as the TOF ratio. [75]. There is a lack of evidence in the current ICU guidelines [32] relating to monitoring neuromuscular blockade. In the postoperative setting, a residual neuromuscular blockade (TOF < 0.9) is still related to a high incidence of unfavorable outcomes such that quantitative monitoring is considered necessary in the intraoperative management of neuromuscular blockade [75], as recommended by the latest French guidelines [76] on muscle relaxants in 2020 and by new European Society of Anesthesia and Intensive Care and American Society of Anesthesiologists guidelines [75,77]. In accordance with these guidelines, the Italian intersociety consensus on perioperative anesthesia care in thoracic surgery recommends strict neuromuscular monitoring for correct administration of both NMBAs and reversal agents [78].

Titration of the level of a neuromuscular blockade based on the patient’s condition (such as renal or hepatic failure) might be considered to avoid prolonged paralysis in the ICU [26]. Residual neuromuscular blockade in the ICU is unrecognized and underreported because monitoring is not commonly carried out in this setting. In a recent study, residual neuromuscular weakness was often considered unrecognized before extubation [79]. A case report described by Workum et al. [80] reported an unusual protracted effect of NMBAs, highlighting the complexity of neuromuscular blockade in the ICU. Thus, monitoring using TOF measurements in the ICU and choosing cisatracurium over rocuronium in critically ill patients should be considered [80].

A recent trial explored the efficacy of TOF monitoring to guide clinical neuromuscular blockade compared with clinical monitoring alone in patients with ARDS. They found no significant change in ICU mortality between the two groups [81]. New research is needed to better assess which is the best NMBA in each clinical situation and how to monitor neuromuscular blockade in the ICU context.

The use of deep sedation and analgesia is always required with NMBAs [32,82]. Patients undergoing MV are often in pain; thus, sedation is necessary to facilitate tolerance to the endotracheal tube, endotracheal suction, and prolonged immobility [83,84]. Strictly sedation monitoring in the ICU could be performed with the bi-spectral index of the electroencephalogram (BIS) or E-entropy, a non-invasive technique easily obtained at the bedside [85]. However, the BIS score is not always considered reliable because of variability in the patient response caused by forehead muscle tone and electrical and mechanical interference, particularly in ICU patients [82]. In this case, NMBAs could be useful to abolish muscle contractions. A small study, conducted by Messner et al. [86], considered the effect of complete muscle relaxation on BIS in fully awake and non-sedated individuals and reported a significant decrease in BIS levels when NMBAs were administered. Other studies showed similar results in sedated patients [87,88]. Even though the use of BIS is advantageous, its systematic use is not recommended in the ICU, [89], and more studies are required to better understand if this monitoring modality is valid for mechanically ventilated patients in the ICU [90].

In summary, the use of NMBAs in patients with lung diseases seems quite safe if the sedative state is adequately monitored [89]. Nevertheless, NMBAs use is still controversial, especially considering the lack of updated guidelines concerning sedation, reversal, and monitoring [31,75,76,90].

## 10. Conclusions

The appropriate use of NMBAs in critically ill patients with lung diseases is unclear, and proper indications for their use are still required, including appropriate timing and careful monitoring of the duration of administration to reduce side effects while allowing for the advantage of their benefits, such as improved oxygenation. The lack of well-designed prospective trials reflects the controversial results.

There is a lack of strong and updated recommendations for the use of NMBAs in the ICU setting. Precise monitoring of the neuromuscular blockade is considered a useful strategy by which to minimize residual weakness and other detrimental effects which are not so rare in the ICU. In this case, TOF might play a role, but its use in the ICU setting is still unclear. Although current knowledge is lacking concerning studies with long-term outcomes conducted on ICU patients, in accordance with the recent guidelines, the administration of NMBAs should be limited to avoid ventilator asynchrony with a personalized approach based on each individual clinical setting. Current knowledge suggests that the use of NMBAs in critically ill patients with lung diseases must be individualized, and further studies are required. Other indications will come from new ongoing clinical trials.

## Figures and Tables

**Table 2:** Randomized trials and metanalysis focusing on the use of NMBAs in patients with ARDS.

Authors/Year	Type of Study/Population	Subtypes of Drugs	Objective	Outcome
Gainnier et al. [10] 2004	Multi-center, prospective, controlled, randomized trial: 56 patients with PaO_2_/FiO_2_ < 150 with PEEP ≥ 5 cm H_2_O randomized in control (*n* = 28) and NMBA (*n* = 28) groups	not specified	Evaluate the effects of a 48 h NMBAs infusion on gas exchange over a 120 h time period in patients with ARDS	NMBAs were administered for 48 h; oxygenation (PaO_2_/FiO_2_ ratio) was better in NMBA compared to control group
Papazian et al. (ACURASYS trial) [54] 2010	Multi-center, prospective, controlled, randomized trial: 340 patients with severe ARDS randomized in placebo (*n* = 162) and NMBA (*n* = 178) groups	cisatracurium	Evaluate clinical outcomes after 48 h of therapy with NMBAs in patients with early, severe ARDS	Early administration of NMBAs for 48 h decreased 90-day mortality (31.6% with NMBAs vs. 40.7% in the placebo group) and risk of barotrauma in patients with moderate to severe ARDS
Forel et al. [14] 2006	Multi-center, prospective, controlled, and randomized trial: 36 patients with PaO_2_/FiO_2_ < 200 at a PEEP ≥ 5 cm H_2_O randomized in placebo (*n* = 18) and NMBA (*n* = 18) groups	cisatracurium	Evaluate the effects of NMBAs on pulmonary and systemic inflammation in patients with ARDS ventilated with a lung-protective strategy	At 48 h after randomization, pulmonary concentrations of IL-1β (*p* = 0.005), IL-6 (*p* = 0.038), and IL-8 (*p* = 0.017) and serum concentration of IL-6 (*p* = 0.05) and IL-8 (*p* = 0.003) were lower in the NMBA group as compared with the control group; improvement in PaO_2_/FiO_2_ ratio was observed and reinforced in the NMBA group (*p* < 0.001)
The National Heart, Lung, and Blood Institute PETAL Clinical Trials Network (ROSE trial) [52] 2019	Multi-center, prospective, controlled, randomized trial: 1006 patients with moderate-to-severe ARDS PaO_2_/FiO_2_ < 150 with PEEP ≥ 8 cm H_2_O, randomized in intervention (*n* = 501) and control (*n* = 505) groups	cisatracurium	Evaluate mortality at 90 days in patients with moderate-to-severe ARDS randomly divided into two groups: the intervention group, treated with 48 h infusion of NMBA with concomitant deep sedation, or the control group (no NMBA)	Mortality rate at 90 days did not differ between groups (42.5% in the control group vs. 42.8% in the intervention group
Lyu et al. [55] 2014	Prospective study: 96 patients randomized into severe ARDS (*n* = 48) and moderate ARDS (*n* = 48) groups according to the Berlin definition of ARDS; patients were than randomly divided into treatment (*n* = 24) and control (*n* = 24) groups	vecuronium	Observe the clinical effects of early use of NMBA in patients with severe sepsis and ARDS	Sepsis scores improved after treatment with NMBAs in severe ARDS group compared with control group (APACHEII score: 16.58 ± 2.41 vs. 19.79 ± 3.52, t = 3.679, *p* = 0.010; SOFA score: 12.04 ± 2.17 vs. 14.75 ± 3.26, t = 3.385, *p* = 0.010; PaO_2_/FiO_2_: 159.31 ± 22.57 mmHg vs. 131.81 ± 34.93 mmHg, t = 3.239, *p* = 0.020; ScvO_2_: 0.673 ± 0.068 vs. 0.572 ± 0.142, t = 3.137, *p* = 0.030; Lac: 3.10 ± 1.01 mmol/L vs. 4.39 ± 1.72 mmol/L, t = 3.161, *p* = 0.030), while the value of CRP showed no significant difference (180.91 ± 37.14 mg/L vs. 174.66 ± 38.46 mg/L, t = 0.572, *p* = 0.570); 21-day mortality in treatment group was significantly lower than that in the control group [20.8% (5/24) vs. 50.0% (12/24), χ(2) = 4.463, *p* = 0.035].
Guervilly et al. [9] 2017	Randomized controlled trial: 30 patients with moderate to severe ARDS; 6 of them were defined as severe ARDS and treated with 7ysatracurium; 24 patients were classified as moderate ARDS; 13/24 treated with 7ysatracurium; 11/24 not treated with NMBA	cisatracurium	Investigate whether NMBA exert beneficial effects in ARDS by reason of their action on respiratory mechanics, particularly transpulmonary pressures (P _L_)	NMBA infusion was associated with an improvement in oxygenation (higher PaO_2_/FiO_2_) in moderate and severe ARDS, accompanied by a decrease in both Pplat and total PEEP; the mean inspiratory and expiratory P _L_ were higher in the moderate ARDS group receiving NMBA than in the control group; no change driving pressure or ∆P _L_ related to NMBA administration
**Meta-analyses**				
Alhazzani et al. [56] 2013	Metanalysis: three trials (431 patients)	cisatracurium	Evaluate mortality effect and risk of ICU-acquired weakness in patients with ARDS treated with neuromuscular blockade	Short-term infusion of 7ysatracurium was associated with lower hospital mortality (RR, 0.72; 95% CI, 0.58 to 0.91); lower risk of barotrauma (RR, 0.43; 95% CI, 0.20 to 0.90); no effect on the duration of MV was reported (MD, 0.25 days; 95% CI, 5.48 to 5.99), or the risk of ICU-acquired weakness (RR, 1.08; 95% CI, 0.83 to 1.41)
Torbic et al. [5] 2021	Metanalysis: six studies (1558 subjects)	not specified	Evaluate differences in mortality comparing subjects with ARDS who received NMBA to those who received placebo or usual care	NMBAs were associated with a reduction in 21 to 28-day mortality (RR = 0.71 [95% CI 0.52–0.98], but not at 90-day mortality RR = 0.81 [95% CI 0.64–1.04])
Chang et al. [55] 2020	Metanalysis: seven trials (1598 patients)	not specified	Evaluate the effects of NMBA use in patients with moderate-to-severe ARDS	Improvement in oxygenation and reduction in barotrauma risk (RR 0.56, 95% CI 0.36 to 0.87); decreasing mortality at 28 days (RR 0.74, 95% CI 0.56 to 0.9) and 90 days (RR 0.77, 95% CI 0.60 to 0.99)
Hua et al. [56] 2020	Metanalysis: six RCTs (1557 patients)	not specified	Evaluate mortality effects of NMBAs on patients with ARDS; the analysis was performed by comparing placebo or NMBAs treatment	Improvement in oxygenation (PaO_2_/FiO_2_ ratio) at 48 h (MD 27.26 mmHg, 95% CI 1.67, 52.84, I^2^ = 92%) and reduction in barotrauma risk (RR 0.55, 95% CI 0.35, 0.85); compared with placebo or usual treatment, NMBAs were associated with lower 21 to 28-day mortality (RR 0.72, 95% CI 0.53–0.97)
Ho et al. [3] 2020	Metanalysis: five RCTs (1461 patients)	cisatracurium	Evaluate NMBAs benefits for Patients with ARDS	The 8ysatracurium group had the same risk of death at 28 days (RR, 0.90; 95% CI, 0.78–1.03; I^2^ = 50%, *p* = 0.12) and 90 days (RR, 0.81; 95% CI, 0.62–1.06; I^2^ = 56%, *p* = 0.06) as the control group (no 8ysatracurium); no differences in MV duration and ventilator-free days; cisatracurium had a significantly lower risk of barotrauma than the control group with no difference in intensive care unit (ICU)-induced weakness; the PaO_2_/FiO_2_ ratio was higher in the 8ysatracurium group but not until 48 h

NMBA, neuromuscular blocking agent; ARDS, acute respiratory distress syndrome; CRP, C-reactive protein; IL, interleukin; PaO_2_/FiO_2_, arterial partial pressure of oxygen/ fraction of inspired oxygen ratio; PEEP, positive end-expiratory pressure; RR, relative risk; CI, confidence interval; ICU, intensive care unit; MV, mechanical ventilation, MD, mean difference; ScvO_2_, central venous saturation of oxygen; RCT, randomized controlled trial.

## Data Availability

Not applicable.

## References

[1] Fierro M.A., Bartz R.R. (2016). Management of Sedation and Paralysis. Clin. Chest Med..

[2] Bourenne J., Hraiech S., Roch A., Gainnier M., Papazian L., Forel J.-M. (2017). Sedation and Neuromuscular Blocking Agents in Acute Respiratory Distress Syndrome. Ann. Transl. Med..

[3] Ho A.T.N., Patolia S., Guervilly C. (2020). Neuromuscular Blockade in Acute Respiratory Distress Syndrome: A Systematic Review and Meta-Analysis of Randomized Controlled Trials. J. Intensive Care.

[4] Renew J.R., Ratzlaff R., Hernandez-Torres V., Brull S.J., Prielipp R.C. (2020). Neuromuscular Blockade Management in the Critically Ill Patient. J. Intensive Care.

[5] Torbic H., Krishnan S., Harnegie M.P., Duggal A. (2021). Neuromuscular Blocking Agents for ARDS: A Systematic Review and Meta-Analysis. Respir. Care.

[6] O’Donnell D.E. (2006). COPD Exacerbations · 3: Pathophysiology. Thorax.

[7] Peters J.I., Stupka J.E., Singh H., Rossrucker J., Angel L.F., Melo J., Levine S.M. (2012). Status Asthmaticus in the Medical Intensive Care Unit: A 30-Year Experience. Respir. Med..

[8] Wilsterman M.E.F., De Jager P., Blokpoel R., Frerichs I., Dijkstra S.K., Albers M.J.I.J., Burgerhof J.G.M., Markhorst D.G., Kneyber M.C.J. (2016). Short-Term Effects of Neuromuscular Blockade on Global and Regional Lung Mechanics, Oxygenation and Ventilation in Pediatric Acute Hypoxemic Respiratory Failure. Ann. Intensive Care.

[9] Guervilly C., Bisbal M., Forel J.M., Mechati M., Lehingue S., Bourenne J., Perrin G., Rambaud R., Adda M., Hraiech S. (2017). Effects of Neuromuscular Blockers on Transpulmonary Pressures in Moderate to Severe Acute Respiratory Distress Syndrome. Intensive Care Med..

[10] Gainnier M., Roch A., Forel J.-M., Thirion X., Arnal J.-M., Donati S., Papazian L. (2004). Effect of Neuromuscular Blocking Agents on Gas Exchange in Patients Presenting with Acute Respiratory Distress Syndrome*. Crit. Care Med..

[11] Savoie-White F.H., Tremblay L., Menier C.A., Duval C., Bergeron F., Tadrous M., Tougas J., Guertin J.R., Ugalde P.A. (2023). The Use of Early Neuromuscular Blockage in Acute Respiratory Distress Syndrome: A Systematic Review and Meta-Analyses of Randomized Clinical Trials. Heart Lung.

[12] Wang W., Xu C., Ma X., Zhang X., Xie P. (2020). Intensive Care Unit-Acquired Weakness: A Review of Recent Progress With a Look Toward the Future. Front. Med..

[13] Fagerlund M.J., Eriksson L.I. (2009). Current Concepts in Neuromuscular Transmission. Br. J. Anaesth..

[14] Forel J.-M., Roch A., Marin V., Michelet P., Demory D., Blache J.-L., Perrin G., Gainnier M., Bongrand P., Papazian L. (2006). Neuromuscular Blocking Agents Decrease Inflammatory Response in Patients Presenting with Acute Respiratory Distress Syndrome*. Crit. Care Med..

[15] Slutsky A.S. (2010). Neuromuscular Blocking Agents in ARDS. N. Engl. J. Med..

[16] Sottile P.D., Albers D., Moss M.M. (2018). Neuromuscular Blockade Is Associated with the Attenuation of Biomarkers of Epithelial and Endothelial Injury in Patients with Moderate-to-Severe Acute Respiratory Distress Syndrome. Crit Care.

[17] Stäuble C.G., Blobner M. (2020). The Future of Neuromuscular Blocking Agents. Curr. Opin. Anaesthesiol..

[18] Hager H.H., Burns B. (2023). Succinylcholine Chloride. StatPearls.

[19] Sparr H.J., Beaufort T.M., Fuchs-Buder T. (2001). Newer Neuromuscular Blocking Agents: How Do They Compare with Established Agents?. Drugs.

[20] Hraiech S., Yoshida T., Annane D., Duggal A., Fanelli V., Gacouin A., Heunks L., Jaber S., Sottile P.D., Papazian L. (2020). Myorelaxants in ARDS Patients. Intensive Care Med..

[21] Reintam Blaser A., Regli A., De Keulenaer B., Kimball E.J., Starkopf L., Davis W.A., Greiffenstein P., Starkopf J. (2019). Incidence, Risk Factors, and Outcomes of Intra-Abdominal Hypertension in Critically Ill Patients—A Prospective Multicenter Study (IROI Study). Crit. Care Med..

[22] Malbrain M.L.N.G., Chiumello D., Cesana B.M., Reintam Blaser A., Starkopf J., Sugrue M., Pelosi P., Severgnini P., Hernandez G., Brienza N. (2014). A Systematic Review and Individual Patient Data Meta-Analysis on Intra-Abdominal Hypertension in Critically Ill Patients: The Wake-up Project. World Initiative on Abdominal Hypertension Epidemiology, a Unifying Project (WAKE-Up!). Minerva Anestesiol..

[23] Pelosi P., Quintel M., Malbrain M.L.N.G. (2007). Effect of Intra-Abdominal Pressure on Respiratory Mechanics. Acta Clin. Belg..

[24] Regli A., Pelosi P., Malbrain M.L.N.G. (2019). Ventilation in Patients with Intra-Abdominal Hypertension: What Every Critical Care Physician Needs to Know. Ann. Intensive Care.

[25] De Laet I.E., Malbrain M.L.N.G., De Waele J.J. (2020). A Clinician’s Guide to Management of Intra-Abdominal Hypertension and Abdominal Compartment Syndrome in Critically Ill Patients. Crit. Care.

[26] Deem S., Lee C.M., Curtis J.R. (2003). Acquired Neuromuscular Disorders in the Intensive Care Unit. Am. J. Respir. Crit. Care Med..

[27] Price D.R., Mikkelsen M.E., Umscheid C.A., Armstrong E.J. (2016). Neuromuscular Blocking Agents and Neuromuscular Dysfunction Acquired in Critical Illness: A Systematic Review and Meta-Analysis. Crit. Care Med..

[28] Adnet F., Dhissi G., Borron S.W., Galinski M., Rayeh F., Cupa M., Pourriat J., Lapostolle F. (2001). Complication Profiles of Adult Asthmatics Requiring Paralysis during Mechanical Ventilation. Intensive Care Med..

[29] Kesler S.M., Sprenkle M.D., David W.S., Leatherman J.W. (2009). Severe Weakness Complicating Status Asthmaticus despite Minimal Duration of Neuromuscular Paralysis. Intensive Care Med..

[30] Bellaver P., Schaeffer A.F., Leitao C.B., Rech T.H., Nedel W.L. (2023). Association between Neuromuscular Blocking Agents and the Development of Intensive Care Unit-Acquired Weakness (ICU-AW): A Systematic Review with Meta-Analysis and Trial Sequential Analysis. Anaesth. Crit. Care Pain Med..

[31] Puthucheary Z., Rawal J., Ratnayake G., Harridge S., Montgomery H., Hart N. (2012). Neuromuscular Blockade and Skeletal Muscle Weakness in Critically Ill Patients: Time to Rethink the Evidence?. Am. J. Respir. Crit. Care Med..

[32] Murray M.J., DeBlock H., Erstad B., Gray A., Jacobi J., Jordan C., McGee W., McManus C., Meade M., Nix S. (2016). Clinical Practice Guidelines for Sustained Neuromuscular Blockade in the Adult Critically Ill Patient. Crit. Care Med..

[33] Cedborg A.I.H., Sundman E., Bodén K., Hedström H.W., Kuylenstierna R., Ekberg O., Eriksson L.I. (2014). Pharyngeal Function and Breathing Pattern during Partial Neuromuscular Block in the Elderly. Anesthesiology.

[34] Awadh Behbehani N., Al-Mane F., D’yachkova Y., Paré P., Fitz Gerald J.M. (1999). Myopathy Following Mechanical Ventilation for Acute Severe Asthma. Chest.

[35] Rodríguez-Blanco J., Rodríguez-Yanez T., Rodríguez-Blanco J.D., Almanza-Hurtado A.J., Martínez-Ávila M.C., Borré-Naranjo D., Acuña Caballero M.C., Dueñas-Castell C. (2022). Neuromuscular Blocking Agents in the Intensive Care Unit. J. Int. Med. Res..

[36] Levy B.D., Kitch B., Fanta C.H. (1998). Medical and Ventilatory Management of Status Asthmaticus. Intensive Care Med..

[37] Blanch L., Villagra A., Sales B., Montanya J., Lucangelo U., Luján M., García-Esquirol O., Chacón E., Estruga A., Oliva J.C. (2015). Asynchronies during Mechanical Ventilation Are Associated with Mortality. Intensive Care Med..

[38] Pham T., Telias I., Piraino T., Yoshida T., Brochard L.J. (2018). Asynchrony Consequences and Management. Crit. Care Clin..

[39] De Haro C., Ochagavia A., López-Aguilar J., Fernandez-Gonzalo S., Navarra-Ventura G., Magrans R., Montanyà J., Blanch L. (2019). Patient-Ventilator Asynchronies during Mechanical Ventilation: Current Knowledge and Research Priorities. ICMx.

[40] Yoshida T., Amato M.B.P., Kavanagh B.P. (2018). Understanding Spontaneous vs. Ventilator Breaths: Impact and Monitoring. Intensive Care Med..

[41] Yoshida T., Fujino Y., Amato M.B.P., Kavanagh B.P. (2017). Fifty Years of Research in ARDS. Spontaneous Breathing during Mechanical Ventilation. Risks, Mechanisms, and Management. Am. J. Respir. Crit. Care Med..

[42] Yoshida T., Uchiyama A., Matsuura N., Mashimo T., Fujino Y. (2013). The Comparison of Spontaneous Breathing and Muscle Paralysis in Two Different Severities of Experimental Lung Injury*. Crit. Care Med..

[43] Wei X., Wang Z., Liao X., Guo W., Qin T., Wang S. (2020). Role of Neuromuscular Blocking Agents in Acute Respiratory Distress Syndrome: An Updated Meta-Analysis of Randomized Controlled Trials. Front. Pharmacol..

[44] Balzer F., Weiß B., Kumpf O., Treskatsch S., Spies C., Wernecke K.-D., Krannich A., Kastrup M. (2015). Early Deep Sedation Is Associated with Decreased In-Hospital and Two-Year Follow-Up Survival. Crit. Care.

[45] Mellott K.G., Grap M.J., Munro C.L., Sessler C.N., Wetzel P.A., Nilsestuen J.O., Ketchum J.M. (2014). Patient Ventilator Asynchrony in Critically Ill Adults: Frequency and Types. Heart Lung.

[46] Conti G., Ranieri V.M., Costa R., Garratt C., Wighton A., Spinazzola G., Urbino R., Mascia L., Ferrone G., Pohjanjousi P. (2016). Effects of Dexmedetomidine and Propofol on Patient-Ventilator Interaction in Difficult-to-Wean, Mechanically Ventilated Patients: A Prospective, Open-Label, Randomised, Multicentre Study. Crit. Care.

[47] Holanda M.A., Vasconcelos R.D.S., Ferreira J.C., Pinheiro B.V. (2018). Patient-Ventilator Asynchrony. J. Bras. Pneumol..

[48] Qadir N., Chang S.Y. (2021). Pharmacologic Treatments for Acute Respiratory Distress Syndrome. Crit. Care Clin..

[49] Bellani G., Laffey J.G., Pham T., Fan E., Brochard L., Esteban A., Gattinoni L., van Haren F., Larsson A., McAuley D.F. (2016). Epidemiology, Patterns of Care, and Mortality for Patients With Acute Respiratory Distress Syndrome in Intensive Care Units in 50 Countries. JAMA.

[50] Tsolaki V., Zakynthinos G.E., Papadonta M.-E., Bardaka F., Fotakopoulos G., Pantazopoulos I., Makris D., Zakynthinos E. (2022). Neuromuscular Blockade in the Pre- and COVID-19 ARDS Patients. JPM.

[51] Tarazan N., Alshehri M., Sharif S., Al Duhailib Z., Møller M.H., Belley-Cote E., Alshahrani M., Centofanti J., McIntyre L., Baw B. (2020). Neuromuscular Blocking Agents in Acute Respiratory Distress Syndrome: Updated Systematic Review and Meta-Analysis of Randomized Trials. ICMx.

[52] (2019). The National Heart, Lung, and Blood Institute PETAL Clinical Trials Network Early Neuromuscular Blockade in the Acute Respiratory Distress Syndrome. N. Engl. J. Med..

[53] Grasselli G., Calfee C.S., Camporota L., Poole D., Amato M.B.P., Antonelli M., Arabi Y.M., Baroncelli F., Beitler J.R., Bellani G. (2023). ESICM Guidelines on Acute Respiratory Distress Syndrome: Definition, Phenotyping and Respiratory Support Strategies. Intensive Care Med..

[54] Papazian L., Forel J.-M., Gacouin A., Penot-Ragon C., Perrin G., Loundou A., Jaber S., Arnal J.-M., Perez D., Seghboyan J.-M. (2010). Neuromuscular Blockers in Early Acute Respiratory Distress Syndrome. N. Engl. J. Med..

[55] Lyu G., Wang X., Jiang W., Cai T., Zhang Y. (2014). Clinical study of early use of neuromuscular blocking agents in patients with severe sepsis and acute respiratory distress syndrome. Zhonghua Wei Zhong Bing Ji Jiu Yi Xue.

[56] Alhazzani W., Alshahrani M., Jaeschke R., Forel J., Papazian L., Sevransky J., Meade M.O. (2013). Neuromuscular Blocking Agents in Acute Respiratory Distress Syndrome: A Systematic Review and Meta-Analysis of Randomized Controlled Trials. Crit. Care.

[57] Chang W., Sun Q., Peng F., Xie J., Qiu H., Yang Y. (2020). Validation of Neuromuscular Blocking Agent Use in Acute Respiratory Distress Syndrome: A Meta-Analysis of Randomized Trials. Crit. Care.

[58] Hua Y., Ou X., Li Q., Zhu T. (2020). Neuromuscular Blockers in the Acute Respiratory Distress Syndrome: A Meta-Analysis. PLoS ONE.

[59] Mefford B., Donaldson J.C., Bissell B.D. (2020). To Block or Not: Updates in Neuromuscular Blockade in Acute Respiratory Distress Syndrome. Ann. Pharmacother..

[60] Plens G.M., Droghi M.T., Alcala G.C., Pereira S.M., Wawrzeniak I.C., Victorino J.A., Crivellari C., Grassi A., Rezoagli E., Foti G. (2024). Expiratory Muscle Activity Counteracts PEEP and Is Associated with Fentanyl Dose in ARDS Patients. Am. J. Respir. Crit. Care Med..

[61] Lee B.Y., Lee S.-I., Baek M.S., Baek A.-R., Na Y.S., Kim J.H., Seong G.M., Kim W.-Y. (2022). Lower Driving Pressure and Neuromuscular Blocker Use Are Associated With Decreased Mortality in Patients With COVID-19 ARDS. Respir. Care.

[62] Li Bassi G., Gibbons K., Suen J.Y., Dalton H.J., White N., Corley A., Shrapnel S., Hinton S., Forsyth S., Laffey J.G. (2022). Early Short Course of Neuromuscular Blocking Agents in Patients with COVID-19 ARDS: A Propensity Score Analysis. Crit. Care.

[63] Fan E., Del Sorbo L., Goligher E.C., Hodgson C.L., Munshi L., Walkey A.J., Adhikari N.K.J., Amato M.B.P., Branson R., Brower R.G. (2017). An Official American Thoracic Society/European Society of Intensive Care Medicine/Society of Critical Care Medicine Clinical Practice Guideline: Mechanical Ventilation in Adult Patients with Acute Respiratory Distress Syndrome. Am. J. Respir. Crit. Care Med..

[64] Qadir N., Sahetya S., Munshi L., Summers C., Abrams D., Beitler J., Bellani G., Brower R.G., Burry L., Chen J.-T. (2024). An Update on Management of Adult Patients with Acute Respiratory Distress Syndrome: An Official American Thoracic Society Clinical Practice Guideline. Am. J. Respir. Crit. Care Med..

[65] Chakraborty R.K., Basnet S. (2022). Status Asthmaticus. StatPearls.

[66] Goh A., Chan P. (1999). Acute Myopathy after Status Asthmaticus: Steroids, Myorelaxants or Carbon Dioxide?. Respirology.

[67] Qiao H., Cheng H., Liu L., Yin J. (2016). Potential Factors Involved in the Causation of Rhabdomyolysis Following Status Asthmaticus. Allergy Asthma Clin. Immunol..

[68] Venkatesan P. (2023). GOLD COPD Report: 2023 Update. Lancet Respir. Med..

[69] Vassilakopoulos T. (2008). Understanding Wasted/Ineffective Efforts in Mechanically Ventilated COPD Patients Using the Campbell Diagram. Intensive Care Med..

[70] Petrof B.J., Legaré M., Goldberg P., Milic-Emili J., Gottfried S.B. (1990). Continuous Positive Airway Pressure Reduces Work of Breathing and Dyspnea during Weaning from Mechanical Ventilation in Severe Chronic Obstructive Pulmonary Disease. Am. Rev. Respir. Dis..

[71] Appleton R., Kinsella J. (2012). Intensive Care Unit-Acquired Weakness. Contin. Educ. Anaesth. Crit. Care Pain.

[72] Scaramuzzo G., Ottaviani I., Volta C.A., Spadaro S. (2022). Mechanical Ventilation and COPD: From Pathophysiology to Ventilatory Management. Minerva Med..

[73] Goligher E.C., Dres M., Fan E., Rubenfeld G.D., Scales D.C., Herridge M.S., Vorona S., Sklar M.C., Rittayamai N., Lanys A. (2018). Mechanical Ventilation–Induced Diaphragm Atrophy Strongly Impacts Clinical Outcomes. Am. J. Respir. Crit. Care Med..

[74] Ali H.H., Utting J.E., Gray C. (1970). Stimulus Frequency in the Detection of Neuromuscular Block in Humans. Br. J. Anaesth..

[75] Thilen S.R., Weigel W.A., Todd M.M., Dutton R.P., Lien C.A., Grant S.A., Szokol J.W., Eriksson L.I., Yaster M., Grant M.D. (2023). 2023 American Society of Anesthesiologists Practice Guidelines for Monitoring and Antagonism of Neuromuscular Blockade: A Report by the American Society of Anesthesiologists Task Force on Neuromuscular Blockade. Anesthesiology.

[76] Brull S.J., Eriksson L. (2020). The French Guidelines on Muscle Relaxants and Reversal in Anaesthesia: The Chain Is Finally Broken and the Soul Is Freed. Anaesth. Crit. Care Pain Med..

[77] Motamed C. (2023). Sugammadex in Emergency Situations. J. Pers. Med..

[78] Piccioni F., Droghetti A., Bertani A., Coccia C., Corcione A., Corsico A.G., Crisci R., Curcio C., Del Naja C., Feltracco P. (2020). Recommendations from the Italian Intersociety Consensus on Perioperative Anesthesa Care in Thoracic Surgery (PACTS) Part 2: Intraoperative and Postoperative Care. Perioper. Med..

[79] Calef A., Castelgrande R., Crawley K., Dorris S., Durham J., Lee K., Paras J., Piazza K., Race A., Rider L. (2023). Reversing Neuromuscular Blockade without Nerve Stimulator Guidance in a Postsurgical ICU—An Observational Study. J. Clin. Med..

[80] Workum J.D., Janssen S.H.V., Touw H.R.W. (2020). Considerations in Neuromuscular Blockade in the ICU: A Case Report and Review of the Literature. Case Rep. Crit. Care.

[81] Rezaiguia-Delclaux S., Laverdure F., Genty T., Imbert A., Pilorge C., Amaru P., Sarfati C., Stéphan F. (2021). Neuromuscular Blockade Monitoring in Acute Respiratory Distress Syndrome: Randomized Controlled Trial of Clinical Assessment Alone or with Peripheral Nerve Stimulation. Anesth. Analg..

[82] Tezcan B., Turan S., Ozgok A., Clinic of Anaesthesiology and Reanimation, Department of Intensive Care, Turkiye Yuksek Ihtisas Training and Research Hospital, Ankara, Turkey, Clinic of Anaesthesiology and Reanimation, Turkiye Yuksek Ihtisas Training and Research Hospital, Ankara, Turkey (2019). Current Use of Neuromuscular Blocking Agents in Intensive Care Units. Turk. J. Anaesthesiol. Reanim..

[83] Puntillo K.A., Arai S., Cohen N.H., Gropper M.A., Neuhaus J., Paul S.M., Miaskowski C. (2010). Symptoms Experienced by Intensive Care Unit Patients at High Risk of Dying*. Crit. Care Med..

[84] Desbiens N.A., Wu A.W., Broste S.K., Wenger N.S., Connors A.F., Lynn J., Yasui Y., Phillips R.S., Fulkerson W. (1996). Pain and Satisfaction with Pain Control in Seriously Ill Hospitalized Adults: Findings from the SUPPORT Research Investigations. Crit. Care Med..

[85] Coleman R.M., Tousignant-Laflamme Y., Ouellet P., Parenteau-Goudreault É., Cogan J., Bourgault P. (2015). The Use of the Bispectral Index in the Detection of Pain in Mechanically Ventilated Adults in the Intensive Care Unit: A Review of the Literature. Pain Res. Manag..

[86] Messner M., Beese U., Romstöck J., Dinkel M., Tschaikowsky A.K. (2003). The Bispectral Index Declines During Neuromuscular Block in Fully Awake Persons. Anesth. Analg..

[87] Inoue S., Kawaguchi M., Sasaoka N., Hirai K., Furuya H. (2006). Effects of Neuromuscular Block on Systemic and Cerebral Hemodynamics and Bispectral Index during Moderate or Deep Sedation in Critically Ill Patients. Intensive Care Med..

[88] Vivien B., Di Maria S., Ouattara A., Langeron O., Coriat P., Riou B. (2003). Overestimation of Bispectral Index in Sedated Intensive Care Unit Patients Revealed by Administration of Muscle Relaxant. Anesthesiology.

[89] Shetty R.M., Bellini A., Wijayatilake D.S., Hamilton M.A., Jain R., Karanth S., Namachivayam A. (2018). BIS Monitoring versus Clinical Assessment for Sedation in Mechanically Ventilated Adults in the Intensive Care Unit and Its Impact on Clinical Outcomes and Resource Utilization. Cochrane Database Syst. Rev..

[90] Bilgili B., Montoya J.C., Layon A.J., Berger A.L., Kirchner H.L., Gupta L.K., Gloss D.S. (2017). Utilizing Bi-Spectral Index (BIS) for the Monitoring of Sedated Adult ICU Patients: A Systematic Review. Minerva Anestesiol.

